# Associations between race and survival in pediatric patients with diffuse large B‐cell lymphoma

**DOI:** 10.1002/cam4.3736

**Published:** 2021-01-27

**Authors:** Karishma Khullar, Jesse J. Plascak, Richard Drachtman, Peter D. Cole, Rahul R. Parikh

**Affiliations:** ^1^ Department of Radiation Oncology Rutgers Cancer Institute of New Jersey New Brunswick NJ USA; ^2^ Department of Biostatistics and Epidemiology Rutgers School of Public Health Piscataway NJ USA; ^3^ Section of Pediatric Hematology and Oncology Rutgers Cancer Institute of New Jersey New Brunswick NJ USA

**Keywords:** diffuse large B‐cell lymphoma, health‐care disparities, non‐Hodgkin lymphoma, race, survival

## Abstract

**Background:**

The purpose of this study was to examine the factors associated with disparities in overall survival (OS) by race in pediatric diffuse large B‐cell lymphoma (DLBCL) patients.

**Methods:**

We evaluated clinical features and survival among patients ≤21 years of age diagnosed with stage I–IV DLBCL from 2004 to 2014 from the National Cancer Database (NCDB) using a multivariable Cox proportional hazards model.

**Results:**

Among 1386 pediatric patients with DLBCL, 1023 patients met eligibility criteria. In unadjusted analysis, Black patients had a significantly higher overall death rate than White patients (HR_Black vs. White_ 1.51; 95% CI: 1.02–2.23, *p* = 0.041). The survival disparity did not remain significant in adjusted analysis, though controlling for covariates had little effect on the magnitude of the disparity (HR 1.46; 95% CI 0.93–2.31, *p* = 0.103). In adjusted models, presence of B symptoms, receipt of chemotherapy, stage of disease, and Other insurance were significantly associated with OS. Specifically, patients with B symptoms and those with Other insurance were more likely to die than those without B symptoms or private insurance, respectively (HR 1.75; 95% CI 1.22–2.50, *p* = 0.002) and (HR 2.56; 95% CI, 1.39–4.73, *p* = 0.0027), patients who did not receive chemotherapy were three times more likely to die than those who received chemotherapy (HR 3.10; CI 1.80–5.35, *p* < 0.001), and patients who presented with earlier stage disease were less likely to die from their disease than those with stage IV disease (stages I–III HR 0.34, CI 0.18–0.64, *p* < 0.001; HR 0.50, CI 0.30–0.82, *p* = 0.006, HR 0.72, CI 0.43–1.13, *p* = 0.152, respectively).

**Conclusions:**

Our results suggest that racial disparities in survival may be mediated by clinical and treatment parameters.

## Graphical abstract

The purpose of this study was to examine the factors associated with disparities in overall survival (OS) by race in pediatric diffuse large B‐cell lymphoma (DLBCL) patients. Black patients had a significantly higher overall death rate than White patients (HR_Black vs. White_ 1.54; 95% CI: 1.01–2.35, *p* =0.044). Our results suggest that racial disparities in survival may be mediated by clinical and treatment parameters.

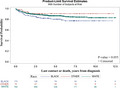

## 1 INTRODUCTION

Research on disparities in nonsolid pediatric tumors has grown in recent years, with several studies finding racial disparities in acute lymphoblastic leukemia (ALL), acute myeloid leukemia (AML), and Hodgkin lymphoma (HL). In pediatric patients with ALL, non‐White patients have been found to have poorer outcomes^1^, ^2^, ^3^, ^4^ with few exceptions.^5^, ^6^ Similarly, in pediatric patients with AML and HL, Black children have been found to have worse survival than their White counterparts.^7^, ^8^, ^9^, ^10^, ^11^, ^12^ While the aforementioned studies indicate the existence of race‐based disparities in childhood cancers, there is also evidence that such disparities may be mediated by socioeconomic status (SES).^13^

Pediatric non‐Hodgkin lymphoma (NHL) represents a clinically and biologically heterogeneous group of diseases and comprises 8% of all childhood malignancies.^14^ Diffuse large B‐cell lymphoma (DLBCL) accounts for 10%–20% of pediatric NHL cases with survival rates of 85–95%.^15^, ^16^ However, unlike studies of adult NHL which have found racial and socioeconomic differences in survival,^17^, ^18^, ^19^, ^20^ studies that investigate racial disparities in pediatric NHL are limited. In studies of NHL in children and young adults, Tai et al. found that being a young adult between the ages of 20 and 29 years was associated with a higher risk of death even after controlling for NHL subtype and stage at diagnosis compared to those <20 years.^21^ Furthermore, while Chao et al. found that Asians had worse survival, Abrahão et al. found that Asians had improved survival relative to their White counterparts, though both studies demonstrated that the survival of those in low SES neighborhoods was adversely impacted.^22^, ^23^ Studies focusing on the subset of pediatric DLBCL are particularly scarce and have focused on pathogenesis and biology rather than racial and socioeconomic disparities. Specifically, pediatric patients with DLBCL have been found to have a predominance of germinal center B‐cell like histology as well as the absence of the translocation *t*(14;18) involving the BCL2 gene and increased c‐Myc protein expression.^16^, ^24^ A better understanding of the impact of racial and socioeconomic disparities on survival in pediatric DLBCL could play an important role in reducing barriers contributing to disparities in care and outcomes and may ultimately help improve survival. In this study, we used the NCDB (National Cancer Database) data on all stages of pediatric DLBCL to examine disparities in overall survival (OS) by race and socioeconomic parameters. We assessed whether OS was impacted by race, age, insurance status, or SES, while adjusting for available confounders. We hypothesized that there may be a racial disparity in survival outcomes in pediatric DLBCL patients and sought to elucidate parameters that may contribute to differences in OS.

## 2 METHODS

### 2.1 Data source

The National Cancer Database (NCDB) is a national, hospital‐based registry sponsored by the American College of Surgeons Commission on Cancer (CoC) and the American Cancer Society. Programs accredited by the CoC report cancers diagnosed or treated in their facilities. The NCDB includes more than 1500 hospital‐based programs and approximately 70% of all newly diagnosed cases in the United States.^25^ The database also contains sociodemographic information including education and income of each patient's zip code of residence at diagnosis as well as individual diagnosis and treatment information such as chemotherapy delivery and radiation therapy (RT) delivery.^26^ Data reporting to the NCDB are highly standardized and the database undergoes extensive internal quality monitoring and validity reviews annually.^25^, ^27^ Approval was sought and obtained from the CoC for the use of the NCDB dataset in this study.

### 2.2 Study population

We considered for inclusion in this study individuals who were 21 years of age and younger within the NCDB diagnosed with their first primary DLBCL between 2004 and 2014 who did not undergo bone marrow transplant (*n* = 1386). We excluded patients who underwent bone marrow transplant because these patients may have had more aggressive disease or primary refractory disease given that transplant is a potential surrogate for relapsed disease.

Covariates considered and categorization were as follows: age (<16 years, 16–19 years, and 19–21 years); race (White, Black, other); ethnicity (Hispanic vs. non‐Hispanic); health insurance at diagnosis (none, private, Medicaid, other); residence‐to‐clinic distance (≤6 miles, 6–19 miles, >19 miles); residential zip code median household income (<$38,000, $38,000–$47,999, $48,000–$62,999, ≥$63,000); residential zip code proportion of residents with <high school diploma (≥21%, 13.0–20.9%, 7.0–12.9%, <7.0%); Charlson‐Deyo comorbidities^28^ (none vs. ≥1); date of diagnosis (<2008, 2008–2011, 2011–2014); Ann Arbor tumor stage at diagnosis (I, II, III, IV); presence of B symptoms (yes vs. no); primary tumor anatomic site (Axilla/Inguinal/Extremity, Chest/Mediastinum, Abdomen/Pelvis, Other/NOS); chemotherapy delivery (none vs. any); RT delivery (none vs. any).

The frequency of unknown or missing values for covariates were as follows: stage (*n* = 169, 12.2%), B symptom status (*n* = 151, 10.1%), vital status (*n* = 109, 7.9%), ethnicity (*n* = 58, 4.2%), race (*n* = 24, 1.7%), health insurance status (*n* = 43, 3.1%), residence‐to‐clinic distance (*n* = 19, 1.4%), radiation delivery (*n* = 13, 0.9%), chemotherapy delivery (*n* = 17, 1.2%), zip code education (*n* = 18, 1.3%), and zip code income (*n* = 18, 1.3%). To investigate the potential for bias from missing data, we tested whether missing covariates varied by race and survival (Supplemental Tables S1 and S2). Because patterns of stage missing data varied by race, we imputed missing stage values using a monotone logistic regression model where missing stage values were predicted from a fitted model.^29^ The fitted model was based on nonmissing stage data predicted by age, race, ethnicity, diagnosis year, comorbidities, receipt of radiation, and receipt of chemotherapy. Missing stage data were assumed to be missing at random, conditional on non‐missing values of variables used in the imputation prediction model above. There was little evidence for any other covariate's missing data varying by race or survival. Thus, the final sample of 1023 consisted of patients with imputed stage and restricted to nonmissing values of all other covariates.

### 2.3 Statistical analysis

Frequencies and proportions were calculated for all variables by race. Chi‐squared tests were used to compare proportions. Kaplan–Meier survival curves and log‐rank statistics were used to examine OS by race. Survival estimates and 95% confidence intervals (CI) were calculated from survival functions at 5 years. Cox proportional hazards regression was used to examine unadjusted hazard ratios (HR) and adjusted hazard (aHR) ratios by race. Survival was calculated in months from the date of diagnosis to the date of last contact or confirmed death. Those not experiencing a study event on 31 December 2016 were right censored. The aforementioned covariates were examined in unadjusted models and were then included in a multivariate model to yield aHRs. Statistical significance was determined at alpha level of 0.05. Results of models based on imputed stage data were analyzed in a multiple imputation framework based on 25 imputed datasets. All statistical analyses were performed using SAS software version 9.4 (SAS Institute).

## 3 RESULTS

### 3.1 Descriptive statistics

Our analysis examined 1023 pediatric patients with stage I‐IV DLBCL who met the inclusion criteria. Patient demographics and clinical parameters are shown in Table 1. The majority of patients in our cohort were White (76.2%), had a disease site not specified (66.5%), received chemotherapy (93.5%), did not receive radiation therapy (81.2%), were privately insured (61.8%), and had no comorbidities (90.5%). Notably, Black patients were more likely than White patients to present with stage IV disease, live in an area where ≥21% did not have a high school diploma, and live in zip codes with a median income <$38,000 (36.1% vs. 27.5%; 38.1% vs. 17.4%; 39.9% vs. 14.1%, respectively).

**TABLE 1:** Covariate frequencies and percent by race among pediatric patients with diffuse large B‐cell lymphoma, 2004–2014, NCDB

Covariate	White	Black	Other	Chi‐square p‐value
Overall	780 (76.2)	173 (16.9)	70 (6.8)	
Age, years				0.101
<16	242 (31.03)	48 (27.75)	29 (41.43)	
16–19	296 (37.95)	58 (33.53)	23 (32.86)	
>19	242 (31.03)	67 (38.73)	18 (25.71)	
Ethnicity				<0.001
Not Hispanic	649 (83.21)	167 (96.53)	67 (95.71)	
Hispanic	131 (16.79)	6 (3.47)	3 (4.29)	
Residential zip code median household income				<0.001
<$38,000	110 (14.10)	69 (39.88)	10 (14.29)	
$38,000‐$47,999	176 (22.56)	42 (24.28)	10 (14.29)	
$48,000‐$62,999	217 (27.82)	37 (21.39)	14 (20.00)	
≥$63,000	277 (35.51)	25 (14.45)	36 (51.43)	
Residential zip code % < high school diploma				<0.001
≥21%	136 (17.44)	66 (38.15)	13 (18.57)	
13.0–20.9%	192 (24.62)	56 (32.37)	16 (22.86)	
7.0–12.9%	243 (31.15)	35 (20.23)	16 (22.86)	
<7.0%	209 (26.79)	16 (9.25)	25 (35.71)	
Health insurance at diagnosis				0.001
None	52 (6.67)	20 (11.56)	8 (11.43)	
Private	510 (65.38)	91 (52.60)	31 (44.29)	
Medicaid	173 (22.18)	58 (33.53)	27 (38.57)	
Other	45 (5.77)	4 (2.31)	4 (5.71)	
Residence‐to‐clinic distance, miles				<0.001
≤6	176 (22.56)	63 (36.42)	21 (30.00)	
>6–19	247 (31.67)	66 (38.15)	23 (32.86)	
>19	357 (45.77)	44 (25.43)	26 (37.14)	
Date of diagnosis				0.557
<2008	266 (34.10)	57 (32.95)	22 (31.43)	
2008–2011	283 (36.28)	71 (41.04)	23 (32.86)	
>2011	231 (29.62)	45 (26.01)	25 (35.71)	
Charlson‐Deyo comorbidities				0.512
None	707 (90.64)	153 (88.44)	66 (94.29)	
≥1	73 (9.36)	20 (11.56)	4 (5.71)	
Anatomic site of primary tumor				0.034
NOS‐Other	511 (65.51)	122 (70.52)	47 (67.14)	
Axilla/Inguinal/Extremity	39 (5.00)	17 (9.83)	2 (2.86)	
Chest/Mediastinum	177 (22.69)	26 (15.03)	14 (20.00)	
Abdomen/Pelvis	53 (6.79)	8 (4.62)	7 (10.00)	
AJCC stage				0.041
I	187 (26.79)	36 (22.78)	14 (24.56)	
II	180 (25.79)	33 (20.89)	23 (40.35)	
III	139 (19.91)	32 (20.25)	6 (10.53)	
IV	192 (27.51)	57 (36.08)	14 (24.56)	
Presence of “B” symptoms				0.08
No	512 (65.64)	98 (56.65)	46 (65.71)	
Yes	268 (34.36)	75 (43.35)	24 (34.29)	
Chemotherapy delivery				0.207
None	53 (6.79)	12 (6.94)	1 (1.43)	
Any	727 (93.21)	161 (93.06)	69 (98.57)	
Radiation delivery				0.794
None	630 (80.77)	143 (82.66)	58 (82.86)	
Any	150 (19.23)	30 (17.34)	12 (17.14)	

### 3.2 Survival analysis

**FIGURE 1:**
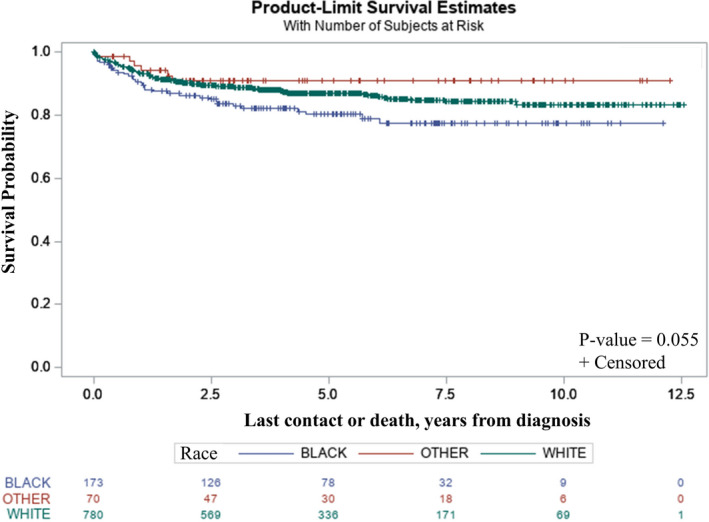
Kaplan–Meier curves of overall survival by race among pediatric patients with diffuse large B‐cell lymphoma, 2004–2014, NCDB

The median follow‐up for the analyzed cohort was 60.2 months. Overall, 5‐year survival was 86.0% (95% CI: 83.6%–88.1%) and there were 140 deaths. Five‐year OS varied by race, but the differences in the survival curves only trended toward significance since the 95% confidence intervals were wide with a log‐rank *p*‐value of 0.055: White OS was 87.0% (95% CI: 84.2%–89.3%), Black OS was 80.2% (95% CI: 72.9%–85.7%), and Other OS was 90.8% (95% CI: 80.8%–95.8%) (Figure 1). In unadjusted analysis, Black patients had a statistically significant overall death rate that was 51% higher than White patients (HR_Black vs. White_ 1.51; 95% CI: 1.02–2.23, *p* = 0.041). Control for available covariates attenuated the significant difference found on univariate analysis since it widened the 95% CI to include the null, but the multivariate analysis had little effect on the magnitude of the all‐cause death disparity comparing Black and White patients (HR 1.46; 95% CI 0.93–2.31, *p* = 0.103). In adjusted models, B symptoms, receipt of chemotherapy, and stage of disease were significantly associated with OS. Specifically, patients reporting the presence of B symptoms were 75% more likely to die than those reporting the absence of B symptoms (HR 1.75; 95% CI 1.22–2.50, *p* = 0.002), patients who did not receive chemotherapy were three times more likely to die than those who received chemotherapy (HR 3.10; CI 1.80–5.35, *p* < 0.001), and patients who presented with earlier stage disease were less likely to die from their disease than those with stage IV disease (stages I‐III HR 0.0.34, CI 0.18–0.64, *p* < 0.001; HR 0.50, CI 0.30–0.82, *p* = 0.006, HR 0.72, CI 0.43–1.13, *p* = 0.152, respectively). Moreover, patients with Other insurance were more likely to die from their disease than those with Private insurance (HR 2.56; 95% CI, 1.39–4.73, *p* = 0.0027). Hazard ratios for all‐cause death by covariates for univariate and multivariate analyses are shown in Table 2. As part of the post hoc sensitivity analyses, we reran the adjusted model using a stage variable dichotomized into earlier (I and II) and later (III and IV). Patients at stage I/II had a hazard of death that was half that of patients at stage III/IV (HR 0.48, 95% CI 0.31, 0.73). No other results appreciably changed.

**TABLE 2:** Hazards of all‐cause death by covariates among pediatric patients with diffuse large B‐cell lymphoma, 2004–2014, NCDB

Covariate	Crude HR (95% CI)	Adjusted HR (95% CI)
Race		
White	1.00	1.00
Black	1.51 (1.02, 2.23)	1.46 (0.93, 2.31)
Other	0.66 (0.29, 1.50)	0.68 (0.29, 1.59)
Age, years		
>19	1.00	1.00
16–19	1.03 (0.69, 1.53)	0.88 (0.56, 1.38)
<16	1.01 (0.67, 1.53)	1.08 (0.71, 1.64)
Ethnicity		
Not hispanic	1.00	1.00
Hispanic	1.10 (0.69, 1.75)	1.02 (0.59, 1.74)
Residential zip code median household income		
≥$63,000	1.00	1.00
$48,000‐$62,999	1.06 (0.65, 1.72)	0.91 (0.54, 1.53)
$38,000‐$47,999	1.10 (0.71, 1.70)	1.00 (0.56, 1.77)
<$38,000	1.08 (0.69, 1.71)	0.79 (0.39, 1.60)
Residential zip code % <high school diploma		
≥21%	1.00	1.00
13.0–20.9%	0.68 (0.43, 1.09)	0.75 (0.45, 1.26)
7.0–12.9%	0.74 (0.47, 1.17)	0.82 (0.46, 1.45)
<7.0%	0.72 (0.45, 1.16)	0.87 (0.44, 1.73)
Health insurance at diagnosis		
Private	1.00	1.00
Medicaid	1.57 (1.08, 2.28)**	1.36 (0.91, 2.03)
Other	2.58 (1.43, 4.65)^***^	2.56 (1.39, 4.73)^***^
None	1.04 (0.52, 2.08)	0.96 (0.47, 1.97)
Residence‐to‐clinic distance, miles		
≤6	1.00	1.00
>6–19	0.81 (0.53, 1.25)	0.87 (0.56, 1.37)
>19	0.87 (0.58, 1.30)	1.01 (0.66, 1.57)
Date of diagnosis		
>2011	1.00	1.00
2008–2011	1.48 (0.92, 2.40)	1.56 (0.95, 2.55)
<2008	1.58 (0.98, 2.57)	1.85 (1.13, 3.02)**
Charlson‐Deyo comorbidities		
None	1.00	1.00
≥1	1.54 (0.94, 2.52)	1.32 (0.79, 2.19)
Anatomic site of primary tumor		
Chest/Mediastinum	1.00	1.00
Axilla/Inguinal/Extremity	0.82 (0.28, 2.42)	0.86 (0.29, 2.58)
Abdomen/Pelvis	2.02 (0.96, 4.25)	1.73 (0.80, 3.73)
NOS‐Other	1.89 (1.16, 3.07)**	1.37 (0.82, 2.29)
AJCC stage		
IV	1.00	1.00
III	0.68 (0.44,1.06)	0.72 (0.45, 1.13)
II	0.43 (0.26,0.69)^***^	0.50 (0.30, 0.82)^***^
I	0.25 (0.14,0.46)^***^	0.34 (0.18, 0.64)^***^
Presence of “B” symptoms		
No	1.00	1.00
Yes	2.06 (1.48, 2.87)^***^	1.75 (1.22, 2.50)^***^
Chemotherapy delivery		
Any	1.00	1.00
None	2.89 (1.78, 4.69)^***^	3.10 (1.80, 5.35)^***^
Radiation delivery		
Any	1.00	1.00
None	1.84 (1.11, 3.05)**	1.39 (0.81, 2.39)

** *p* < 0.05, ^***^ *p* < 0.01.

## 4 DISCUSSION

Our results suggest that the racial disparity in survival may be mediated, in part, by confounding factors that differ by race including presence of B symptoms, stage IV disease, receipt of chemotherapy, and Other insurance given that the racial disparity seen in the univariate models was attenuated in multivariate models and showed that these other variables were significant. Previous studies in adult DLBCL have found certain clinical, biological, and treatment parameters to be associated with survival. Specifically, the presence of B symptoms, lack of receipt of chemotherapy, and more advanced stage have been associated with poorer survival in DLBCL, but the impact of these parameters on race‐based disparities is not as well‐established.^18^, ^30^, ^31^, ^32^, ^33^

Studies regarding racial disparities in pediatric population are scarce, comprise a heterogeneous population of leukemias or lymphomas, and have focused more on the adolescent and young adult (AYA) population. A SEER study of pediatric and AYA patients with leukemia and lymphoma by Viny et al revealed that a racial disparity persisted between Black and White patients between 20 and 29 years of age, but not in the younger age groups.^34^ Chao et al. found that lower income was associated with worse survival and higher mortality in API adolescents which persisted when restricting histology to DLBCL, but not in Black or Hispanic adolescents.^22^ By contrast, Abrahão et al found that Asian patients with DLBCL had a lower risk of death than their White counterparts for reasons which are not well‐understood.^23^ Stage and receipt of chemotherapy have also been found to be important predictors of survival in the AYA population. Tai et al found that pediatric patients and AYAs who were diagnosed with stage III/IV NHL were more likely to die to than those diagnosed with stage I disease, which our study further validates.^21^ Furthermore, receipt and response to chemotherapy has been instrumental in improving survival in NHL over the past few decades. Specifically, Gerard et al found that pediatric and adolescent patients with resected B‐cell NHL who received the adjuvant COPAD chemotherapy regimen had an excellent event‐free survival (EFS) of 98.3% and OS of 99.2%,^35^ while Goldman et al found an EFS and OS of 95% in pediatric and AYA patients with mature B‐cell lymphoma who received rituximab‐based chemotherapy.^36^

We found a racial disparity in survival on unadjusted analysis which dissipated when adjusting for available cofounders. While our study focused solely on pediatric patients rather than adults or AYAs, our results are in accordance with several of the aforementioned studies in that they indicate that clinical and treatment parameters such as B symptoms, stage, as well as receipt of chemotherapy are important predictors in survival and may explain potential observed racial disparities. With regard to SES, we found differences by race with regard to education and income in our sample. In our study, Black patients were disproportionately found to live in an area with a lower median income and where residents did not have a high school diploma compared to their White counterparts which is in agreement with prior studies in pediatric hematologic malignancies.^1^ Insurance was the only SES variable independently associated with survival in our analysis. Similar to prior studies,^37^ we found insurance status to be associated with survival outcomes. Specifically, we found that Other insurance was independently associated with a poorer survival as compared to private insurance, suggesting that survival disparities may be mediated by insurance status, though it is unclear why uninsured patients did not exhibit a poorer survival in the current study. However, unlike previous studies,^22^ we did not find statistically significant associations with regard to income or education on survival outcomes.

The main strength of the current study is its unique focus on a subset of pediatric patients with DLBCL with a representative cohort of Black and White patients. Pediatric NHL is a heterogeneous group of malignancies and several studies have combined different histologic subtypes in their analyses. To our knowledge, our study is the largest dataset examining the role of race on survival in pediatric patients with DLBCL and provides valuable information regarding this potential disparity in this population of patients which should be investigated in future studies.

This study also had several limitations. First, our study may be limited by a small sample size. While our results suggest that the racial disparity found on unadjusted analysis may be mediated by clinical and treatment parameters, it is possible that the small sample size limited our ability to detect race as an independent predictor of survival in multivariate analysis. Nevertheless, our results suggest potentially clinically meaningful differences which should be explored. Second, the study is retrospective and reflective of patients treated in hospital‐based practices. Thus, it may have underrepresented outpatient community practices with more disadvantaged patients that cannot access hospital‐based care. Third, the NCDB does not provide specific information regarding the type of imaging used for staging which could influence prognosis and survival. It also does not provide information regarding receipt of immunotherapy, the response to treatment for either chemotherapy or radiotherapy, or interim PET imaging. Therefore, we were unable to examine the role of a specific intervention on overall survival. Fourth, this database does not include more detailed molecular information of the diagnosed DLBCL, that is, double‐hit versus triple‐hit DLBCL, to allow for further analysis. Finally, while International Prognostic Index is an available variable in the NCDB, it was missing in a high percentage of our dataset, thereby making analysis with this variable unfeasible. Thus, while overall survival may be affected by several factors, our study serves as a descriptive and hypothesis‐generating analysis on potential race‐based disparities in pediatric DLBCL.

In summary, our study found a racial disparity in survival on unadjusted analysis which dissipated when adjusting for available cofounders. Given that the presence of B symptoms, stage at diagnosis, and receipt of chemotherapy were independent predictors of survival, potential racial disparities may be mediated by these clinical and treatment parameters. Our findings are important since they elucidate potential disparities and predictors of survival in a subset of NHL patients for which data is scarce. Further research should focus on better understanding factors that impact survival in this population.

## CONFLICT OF INTEREST

All the authors are responsible for the reported research and have participated in the conception and design, analysis and interpretation of data, and drafting or revising of the manuscript. All the authors have approved the manuscript and agree with this submission. The authors have no conflicts of interest to declare.

## Supporting information

Table S1‐S2

## Data Availability

The data used in the study are derived from a de‐identified NCDB file. The American College of Surgeons and the Commission on Cancer have not verified and are not responsible for the analytic or statistical methodology employed, or the conclusions drawn from these data by the investigator. The NCDB is a joint project of the Commission on Cancer of the American College of Surgeons and the American Cancer Society.
