# Supplementary material for: Associations between race and survival in pediatric patients with diffuse large B‐cell lymphoma
Source: Cancer Med. 2021 Jan 27;10(4):1327–34. doi: 10.1002/cam4.3736 (PMC7926019; doi:10.1002/cam4.3736)
Supplement: Supplementary file 1 — Table S1‐S2 [file CAM4-10-1327-s001.docx]

**SUPPLEMENTARY MATERIAL**

**Table S1. Covariate missing values by race among pediatric patients with Diffuse large B-cell lymphoma, 2004-2014, NCDB**

| **Covariate** | **White** | **Black** | **Other** | **Chi-Square, p-value** |
| --- | --- | --- | --- | --- |
| AJCC Stage |  |  |  | 0.057 |
| Not missing | 907 (88.40) | 212 (87.97) | 76 (80.00) |  |
| Missing | 119 (11.60) | 29 (12.03) | 19 (20.00) |  |
| Presence of "B" Symptoms |  |  |  | 0.230 |
| Not missing | 920 (89.67) | 209 (86.72) | 88 (92.63) |  |
| Missing | 106 (10.33) | 32 (13.28) | 7 ( 7.37) |  |
| Vital Status |  |  |  | 0.890 |
| Not missing | 945 (92.11) | 220 (91.29) | 88 (92.63) |  |
| Missing | 81 ( 7.89) | 21 ( 8.71) | 7 ( 7.37) |  |
| Ethnicity |  |  |  | 0.572 |
| Not missing | 990 (96.49) | 234 (97.10) | 90 (94.74) |  |
| Missing | 36 ( 3.51) | 7 ( 2.90) | 5 ( 5.26) |  |
| Health Insurance at Diagnosis |  |  |  | 0.126 |
| Not missing | 999 (97.37) | 233 (96.68) | 89 (93.68) |  |
| Missing | 27 ( 2.63) | 8 ( 3.32) | 6 ( 6.32) |  |
| Residence-to-clinic distance |  |  |  | 0.205 |
| Not missing | 1017 (99.12) | 236 (97.93) | 93 (97.89) |  |
| Missing | 9 ( 0.88) | 5 ( 2.07) | 2 ( 2.11) |  |
| Radiation Delivery |  |  |  | 0.182 |
| Not missing | 1019 (99.32) | 237 (98.34) | 93 (97.89) |  |
| Missing | 7 ( 0.68) | 4 ( 1.66) | 2 ( 2.11) |  |
| Chemotherapy Delivery |  |  |  | 0.094 |
| Not missing | 1017 (99.12) | 235 (97.51) | 93 (97.89) |  |
| Missing | 9 ( 0.88) | 6 ( 2.49) | 2 ( 2.11) |  |
| Zip code socioeconomic status variables |  |  |  | 0.360 |
| Not missing | 1017 (99.12) | 237 (98.34) | 93 (97.89) |  |
| Missing | 9 ( 0.88) | 4 ( 1.66) | 2 ( 2.11) |  |

**Table S2. Hazards of all-cause death by covariate missing indicators among pediatric patients with Diffuse large B-cell lymphoma, 2004-2014, NCDB^1^**

| **Covariate** | **HR (95% CI)** |
| --- | --- |
| AJCC Stage |  |
| Not missing | 1.00 |
| Missing | 1.21 (0.77,1.89) |
| Presence of "B" Symptoms |  |
| Not missing | 1.00 |
| Missing | 1.24 (0.75, 2.04) |

**^1^** Other covariates with missing data had too few events within those with missing data, preventing modelling of OS by levels of covariate missingness
